# Melanoma in Buckinghamshire: Data from the Inception of the Skin Cancer Multidisciplinary Team

**DOI:** 10.1155/2013/843282

**Published:** 2013-09-16

**Authors:** J. J. Cubitt, A. A. Khan, E. Royston, M. Rughani, M. R. Middleton, P. G Budny

**Affiliations:** ^1^Stoke Mandeville Hospital, Mandeville Road, Aylesbury HP21 8AL, UK; ^2^The Welsh Centre for Burns and Plastic Surgery, Morriston Hospital, Morriston, SA6 6NL, UK; ^3^Oxford NIHR Biomedical Research, Churchill Hospital, Old Road, Headington, OX3 7LE, UK

## Abstract

*Background.* Melanoma incidence is increasing faster than any other cancer in the UK. The introduction of specialist skin cancer multidisciplinary teams intends to improve the provision of care to patients suffering from melanoma. This study aims to investigate the management and survival of patients diagnosed with melanoma around the time of inception of the regional skin cancer multidisciplinary team both to benchmark the service against published data and to enable future analysis of the impact of the specialisation of skin cancer care. *Methods.* All patients diagnosed with primary cutaneous melanoma between January 1, 2003 and December 3, 2005 were identified. Data on clinical and histopathological features, surgical procedures, complications, disease recurrence and 5-year survival were collected and analysed. *Results.* Two hundred and fourteen patients were included, 134 female and 80 males. Median Breslow thickness was 0.74 mm (0.7 mm female and 0.8 mm male). Overall 5-year survival was 88% (90% female and 85% male). *Discussion.* Melanoma incidence in Buckinghamshire is in keeping with published data. Basic demographics details concur with classic melanoma distribution and more recent trends, with increased percentage of superficial spreading and thin melanomas, leading to improved survival are reflected.

## 1. Introduction

Melanoma is currently the 6th commonest cancer in the United Kingdom with more than 12,000 new cases diagnosed each year [1]. The incidence of melanoma is increasing faster than any other cancer in the UK with rises of 62% in males and 49% in females in the last decade. Despite this, the rise in mortality has been more modest with increases of 14% in males and 12% in females, and therefore overall survival rates have improved. This disparity between increased incidence and mortality and improved survival is due to the noncongruent increase in the different histological subtypes: superficial spreading, *in situ*, and thin melanoma incidence has increased significantly more than nodular and thicker melanomas [2, 3]. The prognosis of superficial spreading melanoma is considerably better than nodular melanoma, and therefore survival rates are improving with 80% of men and 90% of women now surviving 5 years [1]. 

The importance of melanoma thickness and depth of invasion for melanoma survival was noted by Clark in 1967 and Breslow in 1970 when they devised their respective staging systems [4]. The thickness of melanoma is still a crucial predictor of melanoma survival and current staging systems, for example, the American Joint Committee on Cancer (AJCC) staging for melanoma, combine the melanoma thickness with pathological information, including ulceration, lymph node metastases, and dermal mitoses (in the most recent edition), to predict the overall survival. The AJCC staging system is recommended by the British Association of dermatologists and the British Association of Plastic and Reconstructive Surgeons and was therefore used in this study. In order to optimise the delivery of skin cancer care in the United Kingdom specialist skin cancer multidisciplinary teams and national melanoma guidelines have been developed [5, 6]. 

The aim of this study was to investigate the surgical management, pathology, and survival outcomes of all patients diagnosed with melanoma who were referred into our regional centre (Buckinghamshire Healthcare NHS Trust) around the time of inception of the regional Skin Cancer Multidisciplinary Team (2003). This is both to benchmark the service against published data and to subsequently be able to gauge the effects of specialisation in provision of skin cancer care. These aims are in line with recommendations from the recent Melanoma Taskforce publication “Quality in Melanoma Care: A Best Practice Pathway” [7].

## 2. Patients and Methods

In 2003, melanoma referrals into the unified skin cancer service of Buckinghamshire Healthcare NHS Trust came through 4 NHS hospitals with a patient catchment population of approximately 500,000 people. Stoke Mandeville Hospital provides the surgical tertiary referral care with complex reconstructions and lymphadenectomies. Nonsurgical oncology for the region is provided by the Churchill Hospital in Oxford and all appropriate patients (stage 2A and above) are referred for consideration of adjuvant treatment or research trials. The skin cancer multidisciplinary team is made up of 5 dermatologists, 2 plastic surgeons, and 2 oncologists. Approximately 2400 new patients are referred into our skin cancer service annually via the “two-week wait” cancer referral system and 11.5% of these are diagnosed and treated for skin cancer.

All patients who received a primary diagnosis of cutaneous melanoma between January 1, 2003 and December 3, 2005 were identified through a histopathological database. Patients with ocular or gynaecological melanoma were excluded along with patients whose initial diagnosis occurred prior to 2003 but had new histological specimens: local recurrence, intransit, nodal or distant metastases during the data collection period. Data on clinical and histopathological features, surgical procedures, complications, disease recurrence (nodal and non-nodal), and 5-year survival were collected. Patients were staged using the 2001 AJCC guidelines as these were used at the time of their diagnosis [8]. Statistical analysis, including Log-rank (Mantel-Cox) test for Kaplan Meier curves and Mann-Whitney test for comparison of medians, was performed using Prism (GraphPad) software and statistical significance was accepted at a *P* value of <0.05. 

## 3. Results 

The results are summarised in Table 1 to aid comparison.

### 3.1. Patient Demographics

**Figure 1:**
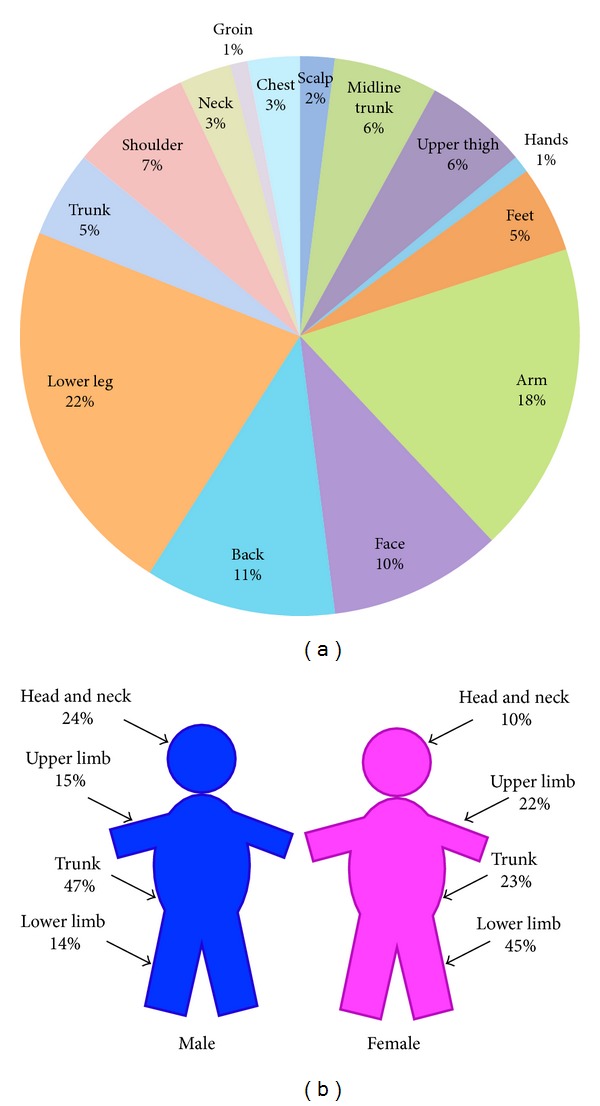
Anatomical distribution of melanomas. (a) Overall (b) Male and Female distribution.

Two hundred and twenty-nine patients were identified, and 214 were included in our final analysis: 134 females (63%) and 80 males (37%). The 15 patients who were excluded moved out of area during the follow-up period. The median age was 62 years (61 years in females and 62 years in males) with an age range from 21 to 100 years. Overall 20% of melanomas arose in high-risk anatomical site (as described by Rogers et al. [7]): hands 1%; scalp 2%, feet 5%, midline trunk 6%, and upper thigh 6% (Figure 1). The commonest site in females was the lower limb (45%) and in men it was the trunk (47%). 

**Figure 2:**
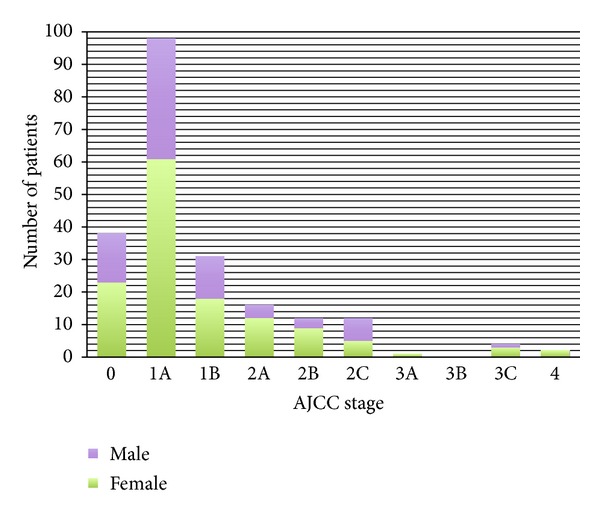
AJCC stage at presentation.

The AJCC stage (2001) at presentation can be seen in Figure 2. There was only 1 patient with a stage 3A at time of initial presentation, as sentinel lymph node biopsies were not routinely carried out at this time. Five out of 214 patients presented with nodal disease (3 cervical, 1 inguinal and 1 axillary) and 3 went on to have lymphadenectomies at the time of their first surgery or treatment margin wider excision. Two patients presented with widespread metastatic disease, 1 of whom had palpable lymphadenopathy and is included in the 5 patients presenting with nodal disease above. Both these patients were referred for oncological management and did not undergo any further surgical management. The remaining 1 patient who presented with lymphadenopathy opted not to have any further management. 

### 3.2. Surgical Excision

The first surgery was performed by a general practitioner in 11% of cases, dermatologists in 72%, plastic surgeons in 16% and a general surgeon in 1% (1 patient with an abdominal melanoma). One hundred and eighty-eight patients went on to have treatment margin wider excision, which was performed by dermatologists in 46%, plastic surgeons in 53%, and an orthopaedic surgeon in 1% (1 patient undergoing toe amputation). 

In general the excision margins for first surgery of suspicious pigmented lesions were 2 mm however in a number of cases where the clinician felt that the lesion was obviously a melanoma, and in discussion with the patient, wider margins were taken to reduce the need for treatment margin wider excision. These margins ranged from 5 mm to 3 cm and this was the case for 23 of the 26 patients who did not undergo secondary excision (5 nodular melanomas, 9 superficial spreading, 5 in situ, 1 lentigo maligna melanoma, 1 acral and 2 cases where the subtype was not specified). The remaining 3 patients who did not undergo treatment margin wider resection were not fit for further surgery due to comorbidities or widespread disease at the time of presentation. Three patients required more than 1 additional excision. 

**Figure 3:**
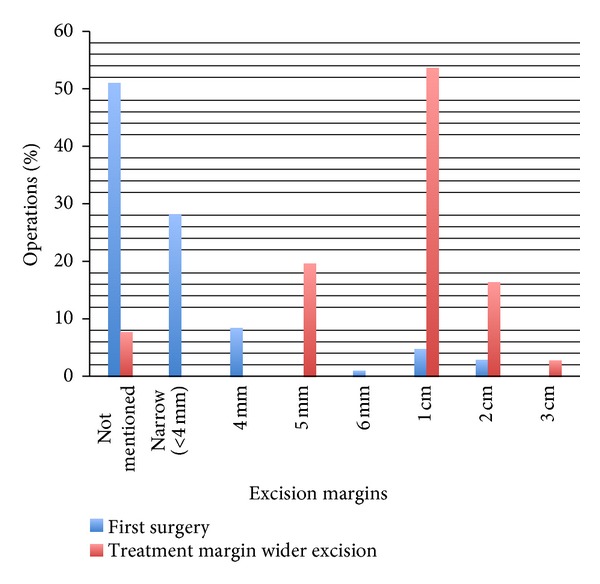
Margins of excision for first and treatment margin wider excision operations.

Fifty-one per cent of first surgery did not have an excision margin documented: 67% of general practitioners, 51% of dermatologists, and 38% of plastic surgeons. Seven percent of treatment margin wider excision also did not have the excision margin documented: representing 8% of dermatologists, and 6% of plastic surgeons. The excision margins for first surgery and treatment margin wider excision are shown in Figure 3. 

Incisional/punch biopsies were carried out in 31 cases, representing 13% of first surgery for general practitioners, 16% of dermatologists and 6% of plastic surgeons. Seven of these lesions were lentigo maligna melanoma, 4 were acral, 10 were superficial spreading, 3 was nodular, 1 in situ and in 6 patients the subtype was not stated. 

The incomplete excision rate for the first surgery, excluding incisional or punch biopsies, was 11%, 24 patients (29% general practitioners, 63% dermatologists, 8% plastic surgeons). Ninety-eight percent of first surgery and 71% of treatment margin wider excisions were closed directly and of the remaining wider excisions, 14% were reconstructed with a local flap, 4% with a full thickness skin graft, and 9% with a split thickness skin graft. 

Histological analysis of the treatment margin wider excision specimens demonstrated residual tumour in 11.7% of these specimens (22 patients). Of these specimens with residual tumour, 6 had clear margins on their primary histology, and in these cases the presence of residual tumour represented microsatellites which correlate to 3% of specimens overall. 

### 3.3. Histology

On histological analysis, 96 patients (45%) had a Breslow thickness of <1 mm, 40 (19%) 1-2 mm, 22 (10%) 2–4 mm, 19 (9%) >4 mm, and 35 (17%) were in situ melanomas. The median Breslow thickness for women was 0.7 mm and for men was 0.8 mm (Mann Whitney Test *P* = 0.7726). 

 Seventeen percent of tumours showed ulceration (20% of female patients and 11% of male), 25% showed regression, 58% showed lymphocytic reaction and 15% showed vascular invasion. 

The melanoma histological subtype observed most commonly was the superficial spreading variety (49%) followed by nodular (17%), in situ (including lentigo maligna, (16%)), lentigo maligna melanoma (5%), acral (2%), and intradermal (1%). The melanoma subtype was not specified in (10%). Nodular melanoma was commoner in females, accounting for (20%) of female melanomas and only (11%) of male melanomas. 

### 3.4. Lymphadenectomy

In our series, a total of 27 patients had nodal disease: 5 at the time of presentation and 22 developing during the follow-up period. Of these, 21 patients went on to have a lymphadenectomy: 5 cervical, 6 axillary and 10 inguinal. The median age of the patients undergoing lymphadenectomy was 69 years and 72 years in the non-operated patients. The average number of nodes harvested was 23 cervical, 9 inguinal and 11 axillary and the mean number of positive melanoma containing nodes were 2 cervical, 3 inguinal and 2 axillary nodes. The 4 patients who presented with lymphadenopathy during follow up but did not have lymphadenectomy included 2 patients with widespread disease and 2 patients who opted not to have any further surgical management.

### 3.5. Survival

#### 3.5.1. Disease Free

**Figure 4:**
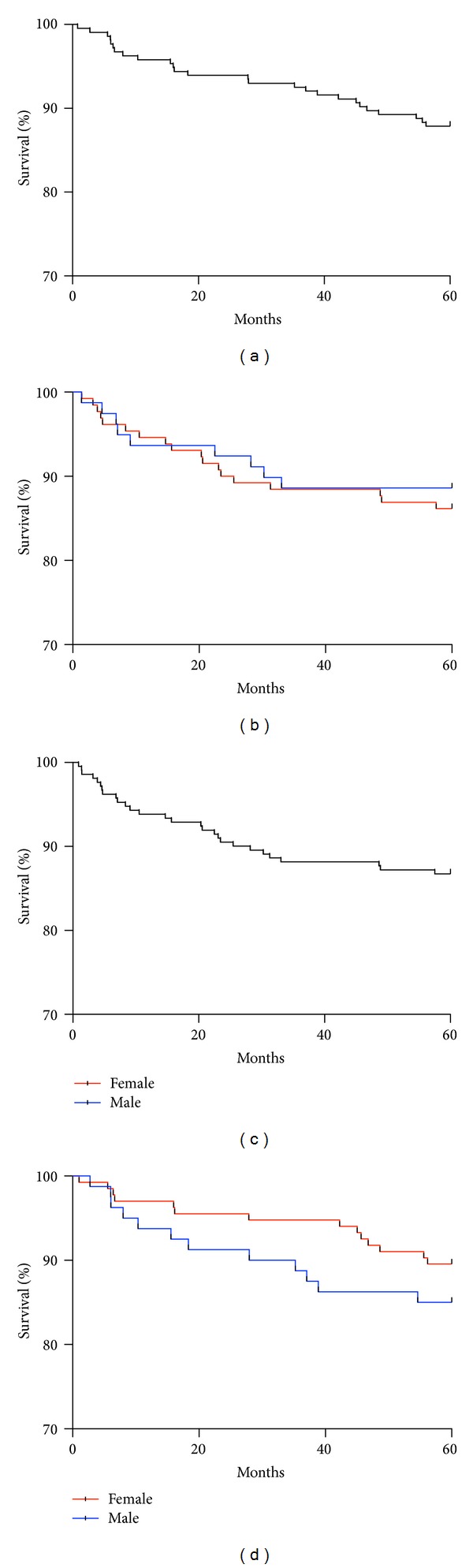
Survival plots over 5 years. (a) Disease free survival overall. (b) Disease free survival by gender. (c) Overall survival. (d) Overall survival by gender.

The 5-year disease-free survival in our series was 87% (86% female and 89% male, *P* = 0.6197) (Figure 4). Twenty-seven out of 214 patients developed nodal metastases and 20 patients developed non-nodal metastases during the follow-up period (9 patients developed both nodal and non-nodal metastases). Of the patients with nonnodal recurrence, 9 developed cutaneous recurrences (7 local and 2 regional) and 7 developed distant visceral metastases. Four out of the 20 patients developed both cutaneous and visceral metastases.

#### 3.5.2. Survival

The overall 5-year survival was 88% (90% female and 85% male, *P* = 0.3021) (Figure 4). Of the patients who died, 9 deaths were melanoma related and 10 were due to other, unrelated, causes, and for 7 the cause of death was unknown.

The survival and recurrence data relating to AJCC staging is displayed in Table 2. 

## 4. Discussion

The overall incidence of melanoma found in our population in Buckinghamshire is 14/100,000 (12/100,000 if melanoma in situ is excluded), which is analogous with the national data and other UK studies [1, 3, 9, 10]. Our population is predominantly Caucasian, and these results are comparable with other predominantly Caucasian populations in Europe [2, 11] and significantly lower than other Caucasian Populations in territories with greater ultraviolet exposure, such as the USA, Australia, and New Zealand [12–14]. 

Our data follows the classical pattern of melanoma data with a female predominance, an average age of 61 years and a higher proportion of lower limb melanoma in women and trunk melanoma in men [10, 15]. The high percentage of superficial spreading melanoma subtype and thin melanoma (lower Breslow thickness) is also in agreement with publications describing the changing trends in histological types and thickness of melanomas being diagnosed globally [4, 16]. This increase in the thin melanoma cohort may reflect screening and increased public awareness of melanoma. 

The majority of first surgery was being performed by specialist doctors which reflects the British Association of Dermatologist guidelines [6]; however significant proportions, 11%, were still being performed by general practitioners. This may partly be due to lesions that are not characteristic of melanoma or due to poor adherence to guidelines. The recent introduction of the Improving Outcomes for People with Skin Tumours by NICE in 2010 has meant that this compliance with the guidelines has significantly improved [17]. All treatment margin wider excisions were carried out by specialist doctors. 

In this cohort we found a high percentage of first surgery and treatment margin wider excisions where the excision margin was not documented. Ideally the excision margin should be documented for all operations so that the width of excision for the wider excision can be planned appropriately. 

On examination of the 188 wider excision specimens, 22 contained residual disease. Six of these patients had clear margins on the primary excision therefore the presence of residual tumour represented the presence of microsatellites. This highlights the importance of wider local excision to ensure the clearance of residual local disease and help guide prognosis [18].

Increasing AJCC stage increases the risk of recurrence and melanoma-related mortality. Our overall survival according to AJCC stage compares favourably with the published national data, however, direct comparison should be limited to stage 1 as the numbers in the other groups are not large enough to give statistical significant results (Table 1) [1, 19]. The national and AJCC data will also include patients who have undergone sentinel lymph node biopsies. If our patients underwent sentinel lymph node biopsies we would expect a number of stage 2 patients to be upgraded to stage 3. This would ultimately improve the outcome of the stage 2 patients. 

The 5-year overall survival for male patients was better than expected (85% as opposed to 80%) which reflects the high proportion of thin melanomas (60% with Breslow thickness <1 mm) and low AJCC stage (65% Stage 0 or 1A). The difference between female and male overall survival (90% and 85%) does not achieve statistical significance (*P* = 0.3021) but raises an interesting point: despite a higher incidence of ulceration (20% versus 11%) and nodular tumours (13% versus 4%), with a very similar Breslow thickness (0.7 mm versus 0.8 mm, *P* = 0.7088) and risk of recurrence within 5 years (14% versus 11%, *P* = 0.6197), women have a greater overall 5-year survival. 

There are some limitations of our data. Patients living in Buckinghamshire may choose to receive melanoma treatment from neighbouring regions or from further afield, closer to their place of work, for example, London. This to some extent would be counteracted by an influx of patients from neighbouring regions but would no doubt exert some mobility bias. In addition, a small number of patients will have chosen to receive treatment in the private sector; however, from available data these numbers appear to be very small. 

We hope that this study acts as a baseline for future comparison of our own service (and others) and will allow evaluation of the impact of specialist skin cancer multidisciplinary teams. Through other studies, we already have an example of early evidence for the value of this development of skin cancer care provision, with the total number of lymph nodes harvested at axillary dissection increasing from a mean of 13.3 in May 2004 to 23.4 in October 2008, reflecting the increasing expertise coming from centralisation of the service. 

## Figures and Tables

**Table 1:** Summary of results.

Variable	Number	Percentage
Total number of patients	214	
Male	80	37%
Female	134	63%
Histology		
In situ	32	15%
Superficial spreading	104	49%
Lentigo maligna	3	1%
Lentigo maligna melanoma	22	5%
Nodular melanoma	36	17%
Acral melanoma	4	2%
Other	2	1%
Not specified	22	10%
Breslow thickness		
In situ	36	17%
<1 mm	96	45%
1-2 mm	40	19%
2–4 mm	22	10%
>4 mm	19	9%
Ulceration		
Present	36	17%
Absent	156	73%
Not Specified	22	10%

**Table 2:** Recurrence and survival by AJCC stage. AJCC stage above 3 is not shown due to the small numbers in the study population.

AJCC stage	5-year disease-free survival (%)	5-year survival (%)	AJCC 5-year survival data (%)	CRUK 5-year survival data (%)
1A	96	97	95	95
1B	81	87	89–91	88–92
2A	69	87	77–79	77–79
2B	73	64	63–67	61–70
2C	53	58	45	43–47
